# Comparison of turbinoplasty surgery efficacy in patients with and without allergic rhinitis^☆^^☆☆^

**DOI:** 10.1016/j.bjorl.2015.10.010

**Published:** 2015-11-06

**Authors:** Rodrigo Hamerschmidt, Rogério Hamerschmidt, Ana Tereza Ramos Moreira, Sérgio Bernardo Tenório, Jorge Rufno Ribas Timi

**Affiliations:** a Department of Otorhinolaryngology, Universidade Federal do Paraná (UFPR), Curitiba, PR, Brazil; b Hospital de Clínicas, Universidade Federal do Paraná (UFPR), Curitiba, PR, Brazil; c Department of Ophthalmology, Universidade Federal do Paraná (UFPR), Curitiba, PR, Brazil; d Department of Anesthesiology, Universidade Federal do Paraná (UFPR), Curitiba, PR, Brazil; e Vascular Surgery, Universidade Federal do Paraná (UFPR), Curitiba, PR, Brazil; f Clinical Surgery, Universidade Federal do Paraná (UFPR), Curitiba, PR, Brazil

**Keywords:** Turbinates, Rhinitis, Olfaction disorders, Smell, Snoring, Nasal obstruction, Conchas nasais, Rinite, Duração da cirurgia, Olfato, Ronco, Obstrução nasal

## Abstract

**Introduction:**

Turbinoplasty is a procedure that aims to reduce the size of the inferior turbinate through exuberant bone removal with high mucosal preservation. The procedure is recommended for patients with or without allergic rhinitis and those showing irreversible hypertrophy of inferior turbinates.

**Objective:**

To evaluate the efficacy of inferior turbinoplasty for obstructive and non-obstructive symptoms in patients with or without allergic rhinitis.

**Methods:**

Prospective study with 57 patients who underwent inferior turbinoplasty. They were evaluated for nasal obstruction, snoring, facial pressure, smell alterations, sneezing, nasal itching and runny nose symptoms, surgery time, and intraoperative bleeding. The last evaluation took place three months after surgery.

**Results:**

Thirty-nine patients with allergic rhinitis and 18 without were assessed. Ninety days after surgery, 94.7% of patients showed degrees IV and V of breathing improvement; 89.5% showed moderate or complete improvement in snoring; all patients showed smell improvement (only one showed moderate improvement; all the others had full improvement); 95.5% experienced complete facial pressure improvement; and 89.7% showed moderate to complete improvement in nasal itching and runny nose symptoms, as well as in sneezing.

**Conclusion:**

The efficacy of inferior turbinoplasty was confirmed not only for obstructive symptoms, but also for non-obstructive symptoms in patients with and without allergic rhinitis.

## Resumo

**Introdução:**

A turbinoplastia é procedimento que visa a redução da concha inferior, à custa da remoção óssea exuberante e maior preservação da mucosa. É indicada para pacientes com e sem rinite alérgica, com hipertrofia irreversível das conchas inferiores.

**Objetivo:**

Avaliar a eficácia da cirurgia de turbinoplastia inferior nos sintomas obstrutivos e não obstrutivos em pacientes com e sem rinite alérgica.

**Método:**

Estudo prospectivo com 57 pacientes submetidos a turbinoplastia inferior. Foram avaliados quanto à obstrução nasal, roncos, pressão facial, alterações no olfato, espirros, prurido nasal e coriza, tempo de cirurgia e sangramento intraoperatório. A última avaliação foi com 3 meses de operação.

**Resultados:**

39 pacientes com rinite alérgica e 18 sem. Com 90 dias de operação, 94,7% dos pacientes apresentaram graus IV e V de melhora na respiração; 89,5% apresentaram melhora moderada ou total dos roncos; todos os pacientes tiveram melhora no olfato (apenas 1 moderada, os demais melhora total); 95,5% obtiveram melhora total da pressão facial e 89,7% obtiveram melhora moderada ou total em prurido nasal, espirros e coriza.

**Conclusão:**

Comprovou-se a eficácia da cirurgia de turbinoplastia inferior não só nos sintomas obstrutivos, mas também nos sintomas não obstrutivos tanto em pacientes com ou sem rinite alérgica.

## Introduction

Nasal obstruction affects approximately 25% of the population.^1^ It is a symptom that can affect people of all ages and ethnicities. It has some degree of morbidity, which varies with the severity and cause of nasal obstruction. The main causes are: septal deviation, inferior and medial turbinate hypertrophy, nasal polyps, and pharyngeal tonsil hypertrophy.^1^ Of all of these alterations, inferior turbinate hypertrophy is the most common cause of nasal obstruction.^2^ The leading causes of inferior turbinate hypertrophy are allergic rhinitis, vasomotor rhinitis, and septal deviation (compensatory hypertrophy).^3^ Bilateral nasal obstruction usually occurs with mucosal disease. When associated with watery rhinorrhea, sneezing, and nasal itching, it is characteristic of nasal mucosa inflammatory edema, especially of allergic nature.^4^

Rhinitis is the inflammation of the nasal mucosal lining, characterized by the presence of one or more symptoms: nasal congestion, rhinorrhea, sneezing, itching, and hyposmia.^5^ Nasal obstruction is one of the most inconvenient symptoms for the patient.^4^ The diagnosis of allergic rhinitis includes personal and family history of atopy, physical examination, and complementary exams. The diagnosis is essentially clinical, taking into account the association of the several symptoms.^5^ The most important complementary exams in the diagnosis of allergic rhinitis, for both specificity and sensitivity, are immediate hypersensitivity skin prick test (SPT) using the puncture technique and evaluation of serum levels of allergen-specific IgE.^5^

The determination of specific IgE *in vitro* may be accomplished by several enzyme immunoassay methods, and more recently, by immunofluorescence. Specific IgE assay *in vitro* for individual allergens, when performed with standardized antigens and adequate technique, has operational characteristics (sensitivity and specificity) that are similar to those of the skin prick test: sensitivity of 89% and specificity of 91%.^5^ Treatment includes both non-pharmacological – environmental control – and pharmacological measures. The latter are based on antihistamines, decongestants, topical and systemic corticoids, and other medications such as ipratropium bromide, chromoglycate disodium, and anti-leukotrienes.

Immunotherapy and the use of saline solution for nasal irrigation are other choices. Modern pharmacology offers many options for clinical treatment of inferior turbinate hypertrophy, whatever the source is. However, although still a controversial issue, most authors agree that when clinical treatment is not sufficient to provide adequate nasal airways, surgical treatment should be indicated.^6^, ^7^

Surgical treatment of allergic rhinitis refractory to clinical treatment is directed to the inferior turbinates and aims to increase the nasal cavity without altering nasal physiology.^5^ The search for effective nasal turbinate treatment has stimulated the surgical skill of rhinologists for over 100 years.^8^

Surgical procedures that aim to reduce mucosal or boney hypertrophy of the inferior nasal turbinate, or both, include: corticosteroid infiltration, lateral dislocation of the nasal turbinate, partial turbinectomy, lower turbinoplasty, cryosurgery, laser vaporization, and radiofrequency. Turbinoplasty is a procedure aimed at reducing the size of the inferior turbinate through exuberant bone removal and meatal surface removal with greater mucosal preservation. It allows reduction of the turbinate volume, while maintaining the physiological functions of the mucosa.^4^ It is performed through an incision along the border of the turbinates, detachment and exposure of the bone surface, followed by bone and redundant mucosa removal, covering the remaining bone with the excess mucosa.^4^ Nasal turbinate surgery is one of the most frequently performed procedures in otorhinolaryngologists’ daily practice, recognized as an effective treatment for nasal obstruction secondary to hypertrophic rhinitis.^9^

Serrano et al. followed 71 patients submitted to inferior turbinate surgery for at least one year, and over 80% attained improvement with the operation.^9^ In 2009, Batra et al. carried out a literature review of 514 studies to verify whether the inferior turbinate surgery improves quality of life, symptoms, and objective parameters with at least six months of follow-up. It was concluded that there is level 4 and 5 evidence of the operation's efficacy in adults with symptomatic hypertrophy of inferior turbinates.^10^ Puterman et al. have performed turbinoplasties since 2002 with excellent results and minimal adverse effects. The procedure removed the lateral mucosa and bone of the inferior turbinate.^11^ The inferior turbinate reduction procedure had a positive impact in patients with persistent allergic rhinitis with clinical treatment.^12^

Mori et al. studied, in a sample of 45 patients, the efficacy of turbinectomy on rhinitis symptoms after five years, and concluded that the operation is a useful strategy to control allergic rhinitis symptoms and helps to improve the quality of life, with significant improvement in symptoms of nasal obstruction (70%) and sneezing (50%).^13^ Mucci et al. in their sample of 55 patients, emphasized the improvement in nasal obstruction in 90% of patients submitted to inferior turbinate surgery, as well as improvement in symptoms such as rhinorrhea, headache, and snoring.^14^ Brandarkar et al. stated that the inferior turbinate surgery was effective and remains the best treatment for hypertrophy unresponsive to medical therapy.^15^

There are several studies in the literature that assessed the improvement in nasal obstruction after the inferior turbinate operation. However, the literature is poor in assessing the impact of turbinate surgery on symptoms such as rhinorrhea, sneezing, and nasal itching.^16^, ^17^ It is also poor in assessing symptoms such as anosmia, snoring, and headache. Few studies have compared postoperative symptoms in patients with and without allergic rhinitis (AR) submitted to inferior turbinate operation.

The aim of this study is to assess the efficacy of inferior turbinoplasty surgery on obstructive and non-obstructive symptoms in patients with and without allergic rhinitis.

## Methods

This longitudinal contemporary cohort study involved 57 patients treated on an outpatient basis, prospectively evaluated and submitted to inferior turbinoplasty surgery from January to December 2013, all performed in the same hospital, after approval by the Research Ethics Committee under No. 0005/2012-05. All patients had inferior turbinate hypertrophy and nasal obstruction refractory to medical treatment with at least two months of topical nasal corticosteroid and systemic antihistamine use, and no other nasal conditions, such as septal deviation, middle concha bullosa, or hypertrophic middle turbinate.

Patients were divided into two groups: group I – patients with nasal obstruction and symptoms of rhinorrhea, sneezing, and itchy nose – were considered as having allergic rhinitis; and group II – patients with nasal obstruction without the other described symptoms – were considered without allergic rhinitis.

Specific IgE assays were performed on the blood of all patients to aeroallergens, carried out preoperatively *in vitro* for detection of allergic rhinitis; the tested allergens, which are common in the local environment, are grasses, dust mites, pollen, fungi, and dog and cat epithelium.

All patients were operated on by the same surgeon, under local anesthesia and sedation.

The following inclusion criteria were used: patients with chronic nasal obstruction, without improvement after standard medications (systemic and topical corticosteroids and systemic antihistamines) for at least two months; aged between 14 and 70 years; willing to participate in the study and to answer the protocol questions; available to return for reassessment seven, 30, and 90 days after surgery; patients with irreversible hypertrophy of the inferior turbinate; patients divided into groups with and without allergic rhinitis, based on the symptoms of itching, sneezing and rhinorrhea, in addition to nasal obstruction.

Patients with any significant anatomical alterations (apart from inferior turbinate hypertrophy) that generated nasal obstruction (septal deviation, middle concha bullosa, nasal valve alterations, nasal tumors of any nasal-sinus origin, retro-nasal or paranasal masses, choanal imperforation, septal perforation, unciform process abnormalities, nasal polyps, adenoid hypertrophy); those who showed improvement after clinical treatment; pregnant women; those who could not undergo the turbinoplasty due to clinical status; and those not willing to participate and answer the protocol questions were excluded.

Patients were evaluated preoperatively regarding gender; age; intensity of nasal obstruction (mild, moderate, severe); presence or absence of snoring; presence or absence of facial pressure; presence or absence of smell alterations; presence or absence of sneezing, itching, and rhinorrhea. Time of operation was assessed transoperatively after turbinate infiltration and placing of cottonoid patties with vasoconstrictor solution until the end of the procedure (0–5 min, 5–10 min, 10–15 min, 15–20 min, over 20 min) and intraoperative bleeding (+/IV; ++/IV; +++/IV; ++++/IV).

At seven days postoperatively, breathing improvement grade (grades 1–5) was assessed. Thirty days after surgery, breathing improvement grade was assessed again. Finally, 90 days after surgery, breathing improvement grade was assessed, as well as olfaction improvement grade (no improvement, moderate improvement, total improvement); facial pressure improvement grade (no improvement, moderate improvement, total improvement); snoring improvement grade (no improvement, moderate improvement, total improvement) and sneezing, itching, and rhinorrhea improvement grade (no improvement, moderate improvement, and total improvement).

**Figure 1:**
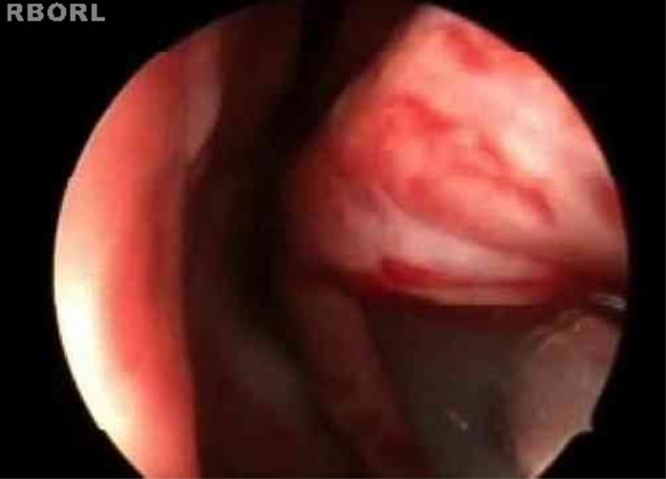
Incision line on the inferior turbinate (endoscopic view).

**Figure 2:**
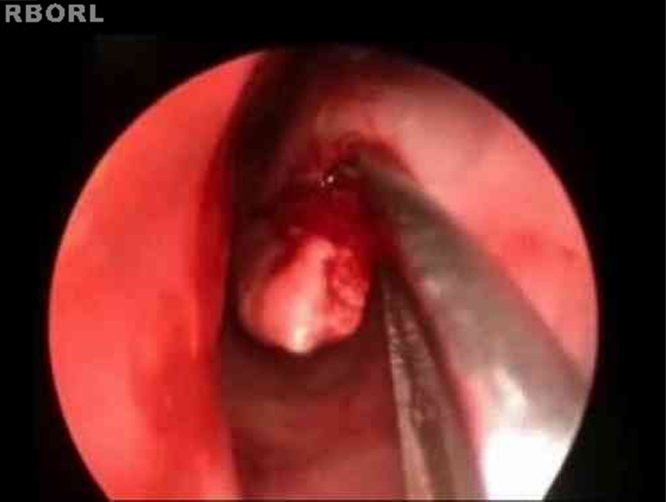
Direction of the turbinectomy scissors blades.

**Figure 3:**
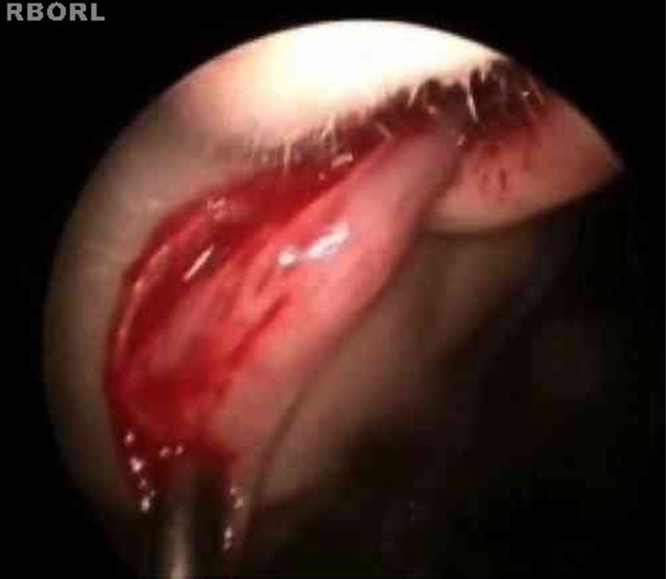
Removal of resected turbinate.

**Figure 4:**
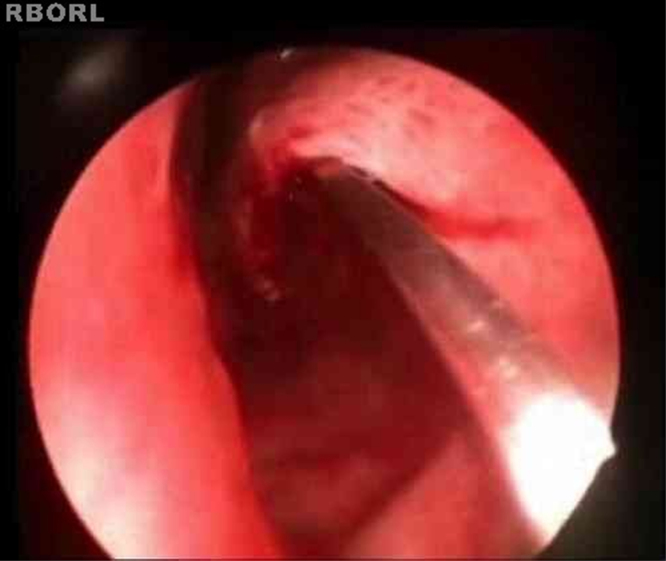
Removal of bony spicule using a chisel.

The turbinoplasty surgery technique consisted of the following: incision in the center of the inferior turbinate in its horizontal extension (Fig. 1); detachment of the entire mucosa above the incision, creating a mucosal flap; next, the turbinate was incised in the anterior–posterior direction using turbinectomy scissors and then one of the scissors blades was directed to the mucosa detached from the bone in the upper part of the turbinate, while the other was directed to the inferior meatus (Fig. 2), thereby removing most of the bone and all its lateral mucosa; the medial mucosa was removed only below the incision, as the mucosa above was used to cover the bony remnant, thereby removing 50% of the medial mucosa (septal surface), 100% of the lateral mucosa (meatal surface), and 70% of the bony turbinate (Fig. 3). Subsequently, the bone spicules were removed with a chisel to reduce the bulging of the remaining turbinate (Fig. 4), and then electrocautery was used to cauterize occasional bleeding spots, particularly in the turbinate tail.

After that, the patients were transferred to the recovery room and discharged in approximately 4 h after the surgery, without nasal packing. They were instructed to avoid efforts or blowing the nose on the first postoperative days and were warned that a small amount of bleeding is common. On the second day after surgery, they started to perform nasal irrigation with 0.9% saline solution to remove the crusts. Saline solution nasal spray could also be used for this purpose. Nasal washing is an important operation step for rapid improvement of nasal obstruction and cleaning.

Statistical analysis of pre-, trans- and postoperative parameters was carried out, comparing the differences between groups I and II. The significance level was set at *p* < 0.05, using the chi-squared and Fisher's exact tests.

## Results

A total of 57 patients were included in this study, of whom 39 were diagnosed with allergic rhinitis (group I) and 18 without (group II). Preoperative data are shown in Table 1. The sample included 30 male individuals, 20 in group I and ten in group II; and 27 females, with 19 in group I and eight in group II.

**Table 1:** Preoperative parameters.

	Gender		Nasal obstruction		Snoring		Facial pressure		Olfaction alterations		IgE	
	Male	Female	Moderate	Severe	Present	Absent	Present	Absent	Present	Absent	Positive	Negative
*With allergic rhinitis*												
*n*	20	19	24	15	29	10	16	23	13	26	29	10
%	51.3	48.7	61.5	38.5	74.4	25.6	41.0	59.0	33.3	66.7	74.4	25.6

*Without allergic rhinitis*												
*n*	10	8	14	4	9	9	6	12	2	16	2	16
%	55.6	44.4	77.8	22.2	50%	50%	33.3	66.7	11.1	88.9	11.1	88.9

*Total*	30	27	38	19	38	19	22	35	15	42	31	26
*p-Value*	0.764		0.227		0.070		0.579		0.077		<0.001	

Age ranged from 14 to 70 years, and there were no statistically significant differences between patients with and without allergic rhinitis (*p* = 0.642).

Nasal obstruction intensity was evaluated in both groups. Most of the patients had moderate to severe complaints in the groups, with no statistically significant differences between them.

As for the presence of preoperative snoring, it was found that most of group I patients snored (29 [74.4%]), whereas nine patients in group II (50%) snored, with no statistically significant differences (*p* = 0.07).

Regarding the presence of facial pressure, 22 patients had the symptom, 16 in group I (41%) and six in group II (33.3%). These numbers did not show statistically significant differences.

The presence of smell alterations was also assessed. Most patients with anosmia/hyposmia (13) were from group I. Only two patients in group II had this alteration. However, these results showed no statistically significant differences.

**Figure 5:**
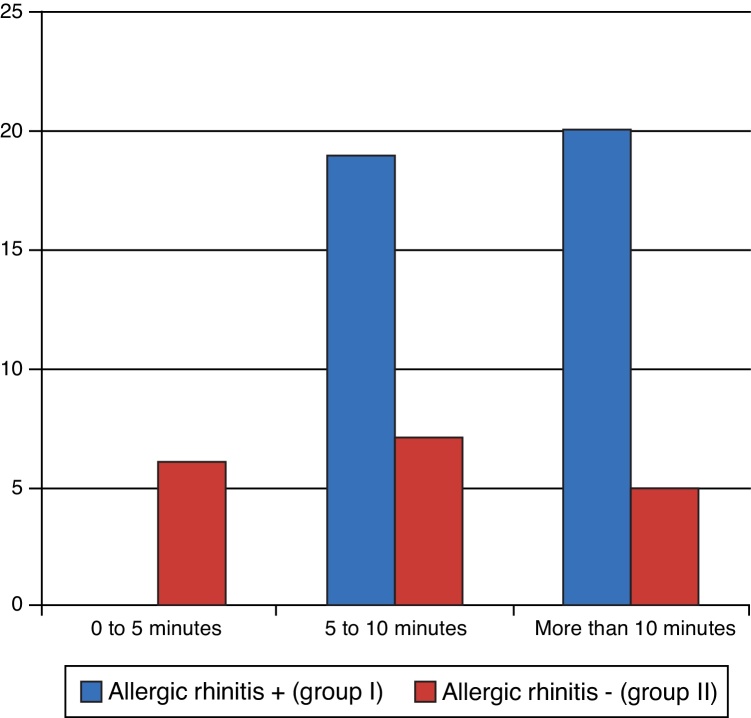
Operative time.

Operating time was assessed in both groups during surgery (Fig. 5). There were statistically significant differences between the groups, as group I had a longer operation time, on average (*p* = 0.001).

**Figure 6:**
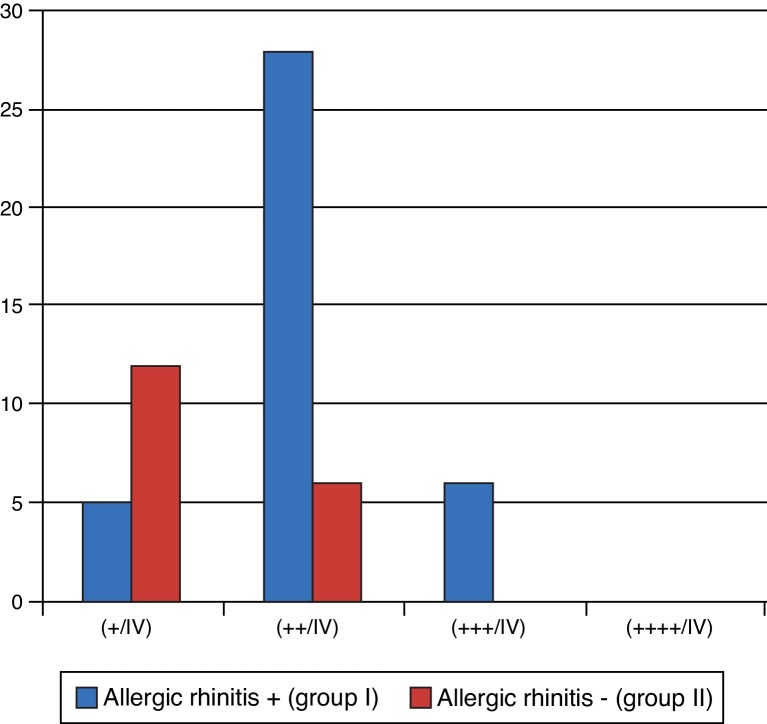
Transoperative bleeding.

Bleeding was also evaluated intraoperatively, which tended to be higher in group I (most degrees + and +++/IV) than in group II (most degrees + and ++/IV) with statistically significant differences (*p* < 0.001), as shown in Fig. 6.

All patients were assessed postoperatively on days seven, 30, and 90.

**Figure 7:**
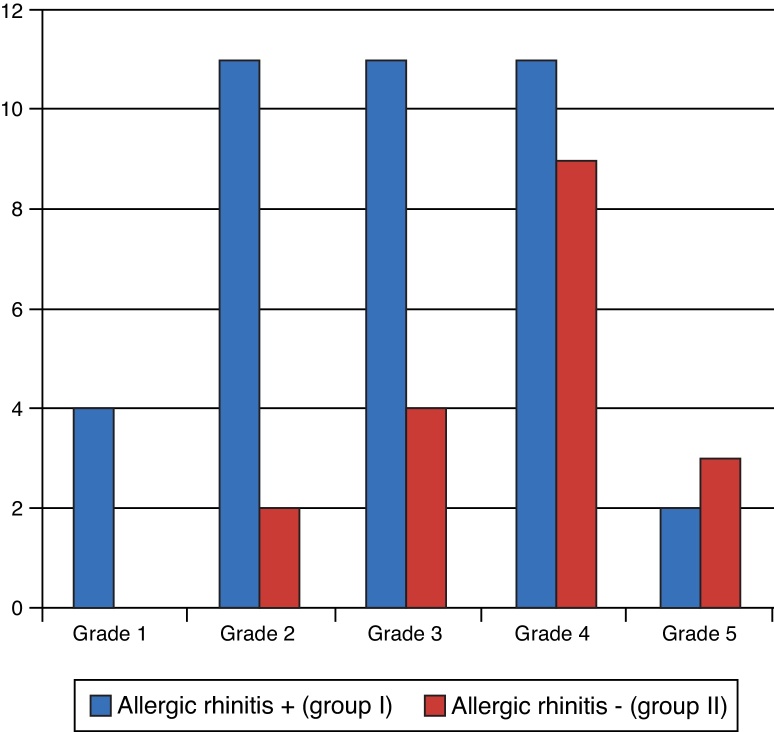
Degree of breathing improvement – seventh day postoperatively.

On the seventh day after the surgery, patients were assessed in relation to breathing. There were no statistically significant differences between groups (*p* = 0.079). The prevalence was higher at grades II, III, and IV (Fig. 7). Improvement was not expected in all patients at this phase, due to swelling and presence of crusts, but many patients already mentioned improvement.

**Figure 8:**
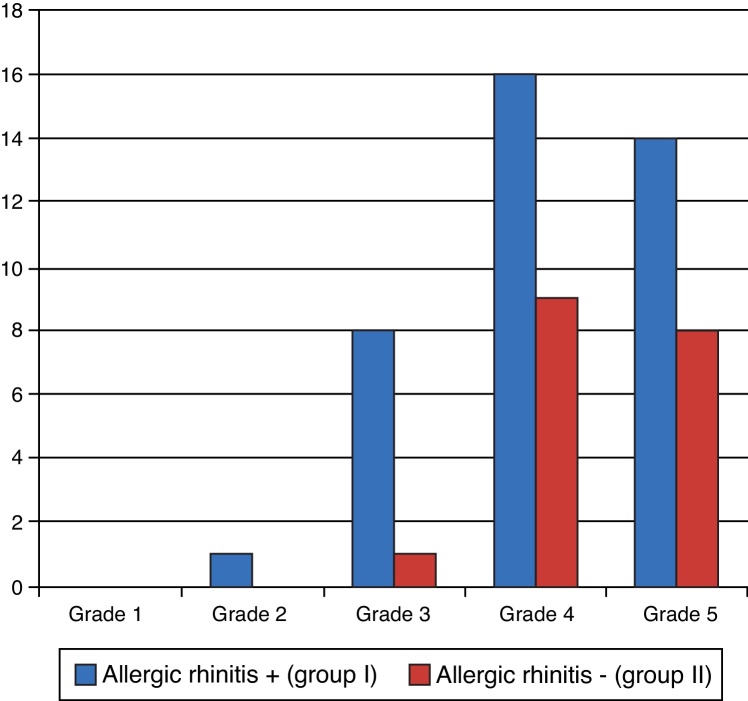
Degree of breathing improvement – 30th day postoperatively.

On the 30th day after surgery, the degree of improvement in breathing was evaluated again and there were still no statistically significant differences between groups (*p* = 0.271), as shown in Fig. 8. Most patients reported improvements at grades IV and V.

**Figure 9:**
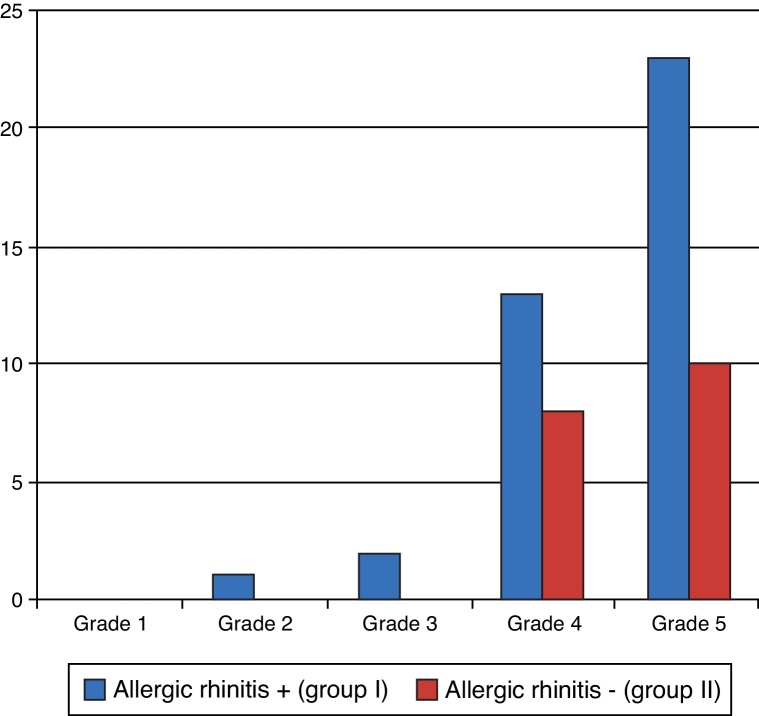
Degree of breathing improvement – 90th day postoperatively.

On the 90th day, the degree of improvement in breathing was reassessed (Fig. 9). This evaluation showed considerable improvement of results, as usually there are no more crusts and edema.^18^ There were no statistically significant differences between the groups (*p* = 0.808).

**Figure 10:**
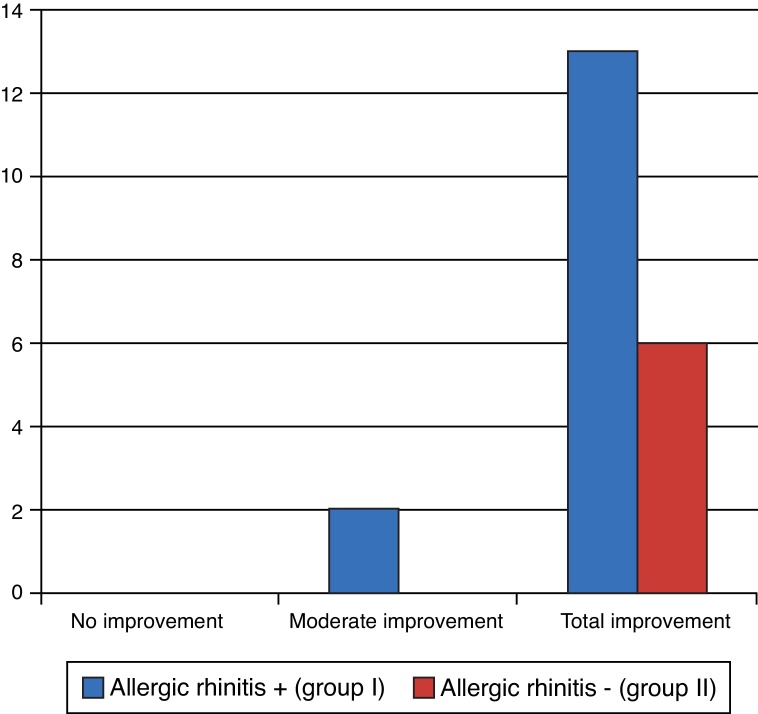
Degree of olfaction improvement.

Regarding the improvement in the smell alteration symptom, all patients showed improvement after the operation. Only two patients in group I showed mild improvement, whereas all others achieved complete improvement (Fig. 10). There were no statistically significant differences (*p* = 1.000 – Fisher's exact test).

**Figure 11:**
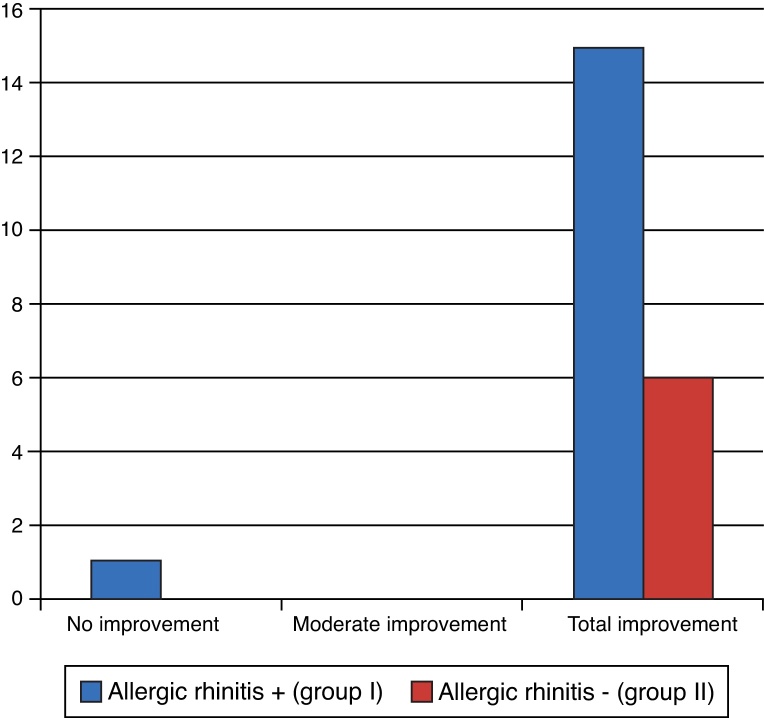
Degree of facial pressure improvement.

Regarding the facial pressure variable, most patients achieved complete improvement with surgery. Only one patient did not achieve any improvement, as shown in Fig. 11. There were no statistically significant differences (*p* = 1.000 – Fisher's exact test).

**Figure 12:**
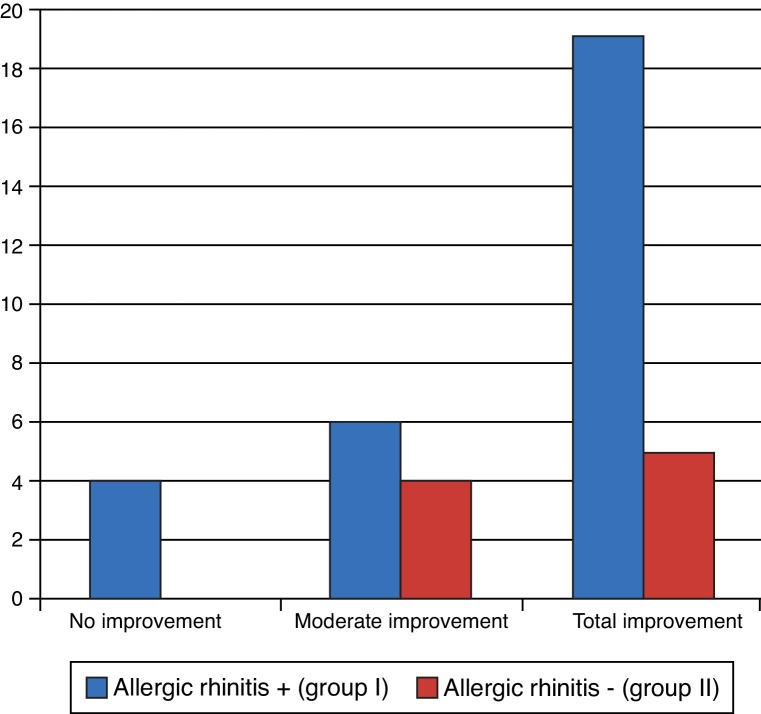
Degree of snoring improvement.

Regarding the snoring, of the 38 patients who snored, most showed moderate or complete improvement, as shown in Fig. 12. There were no statistically significant differences between groups (*p* = 0.588).

**Figure 13:**
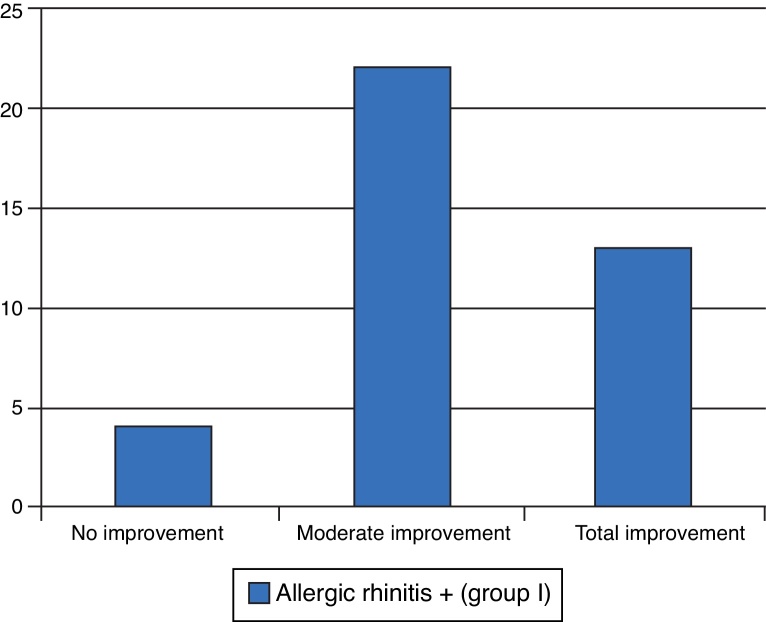
Degree of improvement in sneezing, pruritus, and rhinorrhea.

Finally, on the 90th day postoperatively, improvement in symptoms of sneezing, itching, and rhinorrhea was assessed in group I. Of the 39 patients, only four did not show any improvement, as shown in Fig. 13.

## Discussion

The incidence of rhinitis in adults from the allergy group of the Department of Otorhinolaryngology of Hospital de Clínicas of Faculdade de Medicina of Universidade de São Paulo was 56% for allergic rhinitis and 44% of nonallergic rhinitis. This study only enrolled patients with nasal obstruction. Thus, the incidence of rhinitis in patients with nasal obstruction was much higher. Of the 57 patients, 39 (68.4%) had allergic rhinitis and 18 (31.58%) did not.

Patients with obstructive symptoms who did not achieve any improvement with clinical therapy may benefit from surgical treatment.^9^, ^19^, ^20^, ^21^ Even today, clinical experience shows that the success of the nasal functional operation depends, in many cases, on how the surgeon approaches the nasal turbinates.^21^, ^22^ There are several techniques to reduce the turbinates, and according to Mabry et al., the surgeon must know all the available techniques and use them in each case, as required.^22^

According to the authors’ literature review, the most widely used current procedures are lateral fracture, electrocautery, partial turbinectomy turbinoplasty, and resection with microdebrider. Patients submitted to lower turbinoplasty in the present study had inferior turbinate hypertrophy as the only finding. None of the cases had concomitant septal deviation or another finding, which made it possible for this study to assess the impact of surgery exclusively on the inferior turbinate. None of the postoperative parameters assessed between groups I and II showed statistically significant differences, but all parameters showed improvement in both groups.

Ophir et al. assessed 186 patients at 10–15 years after they had been submitted to inferior turbinate surgery and observed that 88% of patients showed nasal obstruction improvement.^23^ The present study showed similar findings. On the 90th postoperative day, 21 of the 57 patients (36.8%) reported grade IV improvement in breathing, and 33 (57.9%), grade V. Together, 56 (94.7%) of the patients had good improvement in breathing, either grades IV or V. Other rhinitis symptoms than nasal obstruction also show the effect of the nasal surgery. The benefits of surgery on the inferior turbinate are not limited to improvement in nasal obstruction and encompass other rhinitis symptoms, mainly in relation to sneezing.^16^ The present study demonstrated similar findings, as more than 85% of patients who usually had itching, sneezing, and rhinorrhea achieved moderate or complete recovery three months after the surgery. The main objective of inferior turbinate surgery is to allow better circulation of air through the nasal passages. However, as turbinoplasty involves partial turbinate resection, and thus necessarily reduces nasal mucosa surface exposed to the action of allergens and other stimuli, as well as reducing the amount of glandular tissue in this region, this might explain the improvement in other rhinitis symptoms observed in this study. These results demonstrate that the benefits gained from lower turbinoplasty are not limited to nasal obstruction, and patients also can expect improvement in other rhinitis symptoms.

This study also showed an improvement in snoring. Of the 38 patients who snored, 24 (63.16%) reported complete improvement three months after surgery. It is noteworthy that of the 39 patients in group I, 29 (74.4%) snored. Allergic rhinitis is closely related to sleep disorders, and physicians should be aware of this fact, as it represents an unexplored area.^24^ In 2008, Montovani et al. found that sleep disorders were very common in patients with allergic rhinitis (over 90%),^25^ which is in accordance with the findings of the present study.

Facial pressure also showed significant improvement in this study, as of the 22 patients who reported this symptom, 21 (95.5%) showed total improvement, and only one individual (4.55%) from the group with allergic rhinitis showed none. There were no statistical differences between the groups; however, most of them showed improvement. There are no studies on this parameter.

None of the preoperative symptoms showed statistically significant differences between patients with and without allergic rhinitis.

The only finding with statistically significant differences in the preoperative period was the measurement of IgE *in vitro*, in which 29 patients (74.4%) had a positive result in the group with allergic rhinitis and ten (25.6%) did not. In the group without rhinitis, the test was positive in two cases (11.1%). IgE measurement determines that the patient has an allergy, but not necessarily allergic rhinitis.

There are no studies comparing the degree of anosmia improvement with the turbinate operation, but a few studies have reported that the prevalence of anosmia in allergic patients is high. In 2008, Haro et al. emphasized that smell alterations are common in patients with allergic rhinitis.^26^ Olfaction should be investigated in patients with allergic rhinitis through clinical trials, due to the high prevalence of alterations.^27^ Higo et al. investigated smell alterations in patients with allergic rhinitis and speculated that rhinitis causes alterations in the olfactory mucosa, resulting in olfactory transduction impairment.^28^ In the present study, smell alterations in group I were found in 33.3% of individuals (13 patients), and in group II, 11.1% (two patients). Three months postoperatively, only two patients did not achieve overall symptom improvement, with no difference between the two groups.

The assessed transoperative parameters were time of operation and transoperative bleeding. The authors found no studies in the literature that evaluated these parameters in the inferior turbinate surgery between patients with and without allergic rhinitis. These parameters showed statistical differences in the present study. Patients in group I had a longer duration of surgery (*p* = 0.001) and higher transoperative bleeding (*p* < 0.001). One factor could have been the result of the other, considering that when bleeding is higher, the operating time will be longer. This may be important for the scheduling of these surgical patients.

Regarding improvement in breathing, at seven days postoperatively, 40 patients (70.18%) were already in the group with improved grade III, IV, and V. At 30 days, 47 patients (82.5%) reported grades IV and V of improvement, and after three months, this number increased to 54 patients (94.73%). This is consistent with the evolution in the first weeks postoperatively, when edema and crusts may obstruct breathing.

## Conclusion

This study demonstrated the efficacy of inferior turbinoplasty three months after the surgery regarding non-obstructive symptoms of snoring, anosmia, facial pressure, itching, sneezing, and rhinorrhea, in addition to obstructive symptoms in patients with and without allergic rhinitis.

## Funding

This study was supported by 10.13039/501100002322Coordenação de Aperfeiçoamento de Pessoal de Nível Superior (CAPES).

## Conflicts of interest

The authors declare no conflicts of interest.
